# Beyond functional independence: symptom burden and emotional difficulties in pediatric long COVID—a cross-sectional exploratory study

**DOI:** 10.3389/fped.2026.1878494

**Published:** 2026-06-24

**Authors:** Mᵃ Pilar Rodríguez-Pérez, Elisabet Huertas-Hoyas, Sandra León-Herrera, Raquel Gómez-Bravo, Cristina García-Bravo, Esther Rodríguez-Rodríguez, Ana Poveda-García

**Affiliations:** 1 Department of Physical Therapy, Occupational Therapy, Physical Medicine and Rehabilitation of Rey Juan Carlos University, Alcorcón, Spain; 2 Research Group Participation, Roles, Occupations and Activities for the Transformation of Communities, from the Rey Juan Carlos University (PROACT), Rey Juan Carlos University, Alcorcón, Spain; 3 Department of Psychology and Sociology, University of Zaragoza, Zaragoza, Spain; 4 Centre Hospitalier Neuro-Psychiatrique (CHNP), Ettelbruck, Luxembourg; 5 Child and Adolescent Psychiatrist at the Adolescent Crisis and Subacute Care Unit at Fundació Hospitalàries Sant Boi, Barcelona, Spain

**Keywords:** adolescents, children, emotional functioning, functional independence, pediatric long COVID, post-covid condition, school absenteeism

## Abstract

**Background:**

Pediatric Long COVID poses significant challenges to daily functioning, yet its real-world impact remains poorly understood. Standard functional independence measures may not fully capture the condition's consequences in developmentally relevant contexts.

**Methods:**

A cross-sectional exploratory study was conducted with 27 children and adolescents (mean age 15.48 ± 2.31 years; 70.4% female) meeting WHO criteria for Long COVID. Functional independence was assessed using the WeeFIM and emotional functioning with the SDQ. Contextual functioning variables—school attendance, grade repetition, and withdrawal from recreational activities—were collected as exploratory indicators of real-world impact.

**Results:**

Despite high symptom burden—fatigue (81.5%), difficulty concentrating (63.0%), and malaise (55.6%) among the most prevalent, with 88.9% experiencing symptoms for over 24 months—WeeFIM scores were near-ceiling across all domains (total: 114.56 ± 20.71/126). Contextual data revealed substantial real-world impact: only 18.5% attended school regularly, 11.1% had repeated an academic year, and 85.2% had withdrawn from previously enjoyed activities. SDQ total scores fell within the normal range (12.07 ± 5.04), though emotional symptoms were slightly elevated (5.59 ± 2.34). A significant negative correlation was found between SDQ total score and WeeFIM cognition (rho = −0.570, *p* = 0.0019), and a coherent brain fog–mood–concentration symptom cluster was identified. These results are preliminary, hypothesis-generating findings from a convenience sample and should be interpreted with caution regarding generalizability.

**Conclusions:**

Children and adolescents with Long COVID may maintain basic functional independence while experiencing significant symptom burden, emotional difficulties, and substantial restrictions in school, recreational, and social domains. These findings highlight the limited sensitivity of standard functional measures and underscore the need for more comprehensive, context-sensitive assessment approaches in pediatric Long COVID practice.

## 1 Introduction

Since the onset of the COVID-19 pandemic, increasing attention has been directed toward the long-term consequences of SARS-CoV-2 infection, particularly in vulnerable populations such as children and adolescents. Long COVID, also referred to post-COVID condition, describes a set of symptoms that persist for weeks, months or years after the acute phase of SARS-CoV-2 infection. While much of the scientific literature initially focused on the effects of this condition in adults, increasing attention has recently been directed toward its impact on the pediatric population (1). Although children and adolescents generally present with milder clinical manifestations during the acute phase of infection, they may experience persistent consequences that affect multiple domains of daily life (1).

Reported symptoms in pediatric Long COVID include severe fatigue, cognitive difficulties including brain fog (defined as a subjective experience of mental sluggishness, difficulty concentrating, and cognitive fatigue) sleep disturbances, respiratory problems, and, in some cases, emotional and psychological alterations (2). Prospective cohort data confirm that these symptoms can persist for twelve months or beyond in a significant proportion of affected children (3). These effects not only compromise physical well-being but also interfere with broader aspects of daily functioning, including social engagement, academic performance, and involvement in age-appropriate activities that are fundamental for healthy development and adaptation to the environment (4).

Research on pediatric Long COVID has expanded in recent years; however, important conceptual and methodological gaps remain. Much of the literature has focused on symptom prevalence, risk factors, and health-related quality of life, However, how persistent symptoms translate into limitations in real-life, developmentally relevant contexts remains insufficiently understood (5). This is particularly relevant in pediatrics, where the consequences of chronic conditions extend beyond symptom burden and may affect academic engagement, recreation, social involvement, cognitive and emotional development (4).

In rehabilitation and pediatric disability research, it is important to distinguish between functional independence and broader aspects of functioning. Functional independence generally refers to the degree of assistance required to perform daily tasks, whereas functioning in real-life contexts involves the ability to sustain engagement in activities across home, school, and community settings, as conceptualized by the International Classification of Functioning, Disability and Health (ICF) (6). These constructs are related but not interchangeable. A child may remain largely independent in basic activities of daily living while simultaneously experiencing difficulties in maintaining school attendance, leisure engagement, or social involvement. This distinction may be particularly relevant in pediatric Long COVID, where symptoms are often fluctuating, invisible, and highly context-dependent (7).

In conditions characterized by fluctuating, non-visible, and multidimensional symptomatology, such as Long COVID, functional independence alone may not fully capture the real-life impact of the condition. Children and adolescents may retain autonomy in basic daily activities while simultaneously experiencing difficulties in sustaining cognitive effort, managing fatigue, or maintaining consistent engagement in more demanding activities. Consequently, relying exclusively on traditional measures of independence may lead to an underestimation of the functional impact of the condition (7–9).

Traditional functional measures used in pediatric rehabilitation, such as the Functional Independence Measure for Children (WeeFIM), are valuable for assessing autonomy in self-care, mobility, and cognition-related daily tasks. However, these instruments may be less sensitive to the broader impact of post-viral conditions characterized by fatigue, post-exertional worsening, or cognitive overload. In such contexts, preserved independence in basic activities should not be interpreted as equivalent to preserved overall functioning or well-being, but may instead reflect the limited sensitivity of assistance-based measures to capture higher-order difficulties in everyday life (7, 10). An adolescent may dress, bathe, and move around independently while simultaneously accumulating school absences, withdrawing from extracurricular activities previously enjoyed, or experiencing a significant reduction in social engagement, consequences that conventional independence measures are not designed to detect.

Despite their clinical relevance, the assessment of real-life everyday functioning in pediatric Long COVID faces an important methodological challenge: the absence of validated instruments specifically developed for this population. Available participation measures in pediatric rehabilitation, such as the Participation and Environment Measure for Children and Youth (PEM-CY) or the Children's Assessment of Participation and Enjoyment (CAPE/PAC), were developed primarily for children with physical or neurodevelopmental disabilities, and have not been validated for fluctuating post-viral conditions such as Long COVID, whose impact on functioning may be highly variable across times of day, days of the week, and activity contexts (8). This methodological gap is itself a relevant finding regarding the current state of the field and justifies the use of alternative exploratory approaches in early-phase studies.

At the same time, emotional functioning appears to be a clinically relevant dimension of pediatric Long COVID. Recent studies have reported increased anxiety, depressive symptoms, sleep problems, and reduced quality of life in affected children and adolescents, even when overt behavioural problems are not prominent (5, 11). Emotional distress may both result from and exacerbate persistent physical and cognitive symptoms, contributing to a cycle of reduced activity tolerance, academic difficulties, and psychosocial strain that directly affects daily life (12).

Understanding how functional independence, emotional functioning, and real-life everyday functioning interact is essential for developing comprehensive approaches tailored to the specific needs of this population. Effective therapeutic strategies must be grounded in a clear understanding of how persistent symptoms influence not only basic functioning but also emotional well-being and the capacity to remain engaged in meaningful daily routines and activities (4, 13).

Against this background, the present study was designed as an exploratory cross-sectional analysis of children and adolescents with Long COVID, with the aim of examining the relationship between persistent symptom burden, emotional difficulties, and functional independence. Complementarily, and given the absence of validated instruments specifically designed for this population, contextual functioning variables were collected (including school absences, grade repetition, and withdrawal from extracurricular activities) as exploratory indicators of the condition's impact on daily life in developmentally relevant domains. The study seeks to contribute to a more comprehensive understanding of the multidimensional impact of pediatric Long COVID and to provide preliminary evidence to inform both clinical practice and the development of assessment instruments specific to this condition.

## 2 Materials and methods

### 2.1 Study design

A cross-sectional descriptive study was conducted to analyze the impact of Long COVID on functional independence and emotional functioning in children and adolescents. Quantitative methods were employed to examine functional performance across daily activities, including self-care, mobility, and cognition-related tasks, as well as emotional and behavioral difficulties and contextual functioning indicators.

The study was approved by the Ethics Committee of Universidad Rey Juan Carlos N.° 031220246232024. All procedures were conducted in accordance with the Declaration of Helsinki and applicable national data protection regulations (Organic Law 3/2018).

### 2.2 Participants

The sample included children and adolescents who met the World Health Organization (WHO) criteria for Long COVID., defined as the persistence of multisystem symptoms lasting at least two months and occurring three months after the onset of acute SARS-CoV-2 infection, with no alternative medical explanation.

Participants were eligible for inclusion if they were between 10 and 19 years of age, had a diagnosis of Long COVID according to WHO criteria, and presented persistent symptoms at the time of assessment. Exclusion criteria included the presence of pre-existing medical conditions or comorbidities that could interfere with the interpretation of functional outcomes, severe psychiatric disorders or mental health conditions that could compromise reliable participation in the survey, and limited technological access or communication barriers preventing completion of the online assessment.

This study represents an exploratory phase of a broader research project examining the functional and emotional impact of pediatric Long COVID. Due to the specific diagnostic criteria and recruitment through specialized support networks, the final sample consisted of 27 participants. No formal sample size calculation was performed, consistent with the exploratory nature of the study and in line with precedent in early-phase pediatric Long COVID research reporting comparable sample sizes (14). Given the emerging and relatively under-identified nature of pediatric Long COVID, the present findings should be interpreted as preliminary and hypothesis-generating.

Participants were recruited using convenience sampling through national Long COVID patient associations, support networks, and collaborating clinical contacts. Neither consecutive not random sampling was feasible given the under-identified nature of this population. The survey link was distributed anonymously though patient associations' channels, which did not allow tracking of how many families received the invitation or the overall response rate. As recruitment relied on voluntary participation, the sample may have disproportionately attracted families experiencing more persistent symptoms, greater functional impact, or a higher level of engagement with Long COVID advocacy and support initiatives.

### 2.3 Variables and instruments

The primary dependent variables of this study were functional independence and emotional functioning.

Functional independence was assessed using the Functional Independence Measure for Children (WeeFIM), a widely used instrument in pediatric rehabilitation settings. The WeeFIM consists of 18 items grouped into three domains: self-care, mobility, and cognition/social functioning. Each item is scored on a 7-point scale ranging from 1 (total assistance) to 7 (complete independence), with higher scores indicating greater functional autonomy. The instrument has demonstrated strong reliability and validity across pediatric populations and is commonly used to determine the level of assistance required in daily activities (15). The WeeFIM has demonstrated strong psychometric properties, with intraclass correlation coefficients (ICCs) for inter-rater reliability ranging from .95 to .99 across domains, and test-retest reliability ICCs above .90 (10, 15). Concurrent validity has been established through significant correlations with the Pediatric Evaluation of Disability Inventory (PEDI) and other functional measures used in pediatric rehabilitation settings. Validated adaptations are available for Spanish-speaking populations, supporting its applicability in the present study.

The selection of the WeeFIM for a predominantly adolescent sample (mean age 15.48 years; range: 10–19) requires explicit justification. Although the WeeFIM was originally normed for younger pediatric populations, it has been applied in adolescent samples within the pediatric rehabilitation literature and retains clinical utility for evaluating the level of assistance required in self-care, mobility, and cognition-related tasks across the full 0–21-year range for which it is designed (10, 15). In the present study, the WeeFIM was selected as the most widely used standardized functional independence instrument with Spanish-language validation and established clinical precedent in pediatric rehabilitation settings. Alternative participation-based instruments such as the PEM-CY or CAPE/PAC were considered but not adopted as primary outcome measures because they were developed for children with stable physical or neurodevelopmental disabilities and have not been validated for post-viral, fluctuating conditions such as Long COVID. In this exploratory context, the WeeFIM was therefore the most suitable standardized instrument available, while its known limitations in capturing higher-order participation are explicitly acknowledged throughout.

It should also be noted that near-ceiling WeeFIM scores were anticipated *a priori* in this population. Existing literature on pediatric Long COVID consistently documents preserved basic functional independence in children and adolescents despite substantial symptom burden, suggesting that the instrument's ceiling is likely to be approached in this sample (16). This anticipated ceiling effect was therefore not treated as an unexpected finding but rather as a methodologically informative result—one that reinforces the study's central argument that standard independence-based measures are insufficient to capture the full real-life impact of pediatric Long COVID and that more sensitive, context-specific assessment approaches are needed.

Emotional and behavioral functioning was evaluated using the Strengths and Difficulties Questionnaire (SDQ) (17), a widely used screening tool for child and adolescent mental health. The SDQ comprises five subscales assessing emotional symptoms, conduct problems, hyperactivity/inattention, peer relationship problems, and prosocial behavior. The SDQ has been translated into more than 80 languages and validated in Spanish populations. The Spanish validation of the SDQ has reported adequate internal consistency, with Cronbach's *α* values ranging from .54 to .73 across subscales and .79 for the total difficulties score (18). Convergent validity has been demonstrated through significant correlations with the Child Behavior Checklist (CBCL) and other standardized child mental health screening tools across diverse cultural and linguistic contexts. In the present study, scores were interpreted using established Spanish cut-off points, allowing classification of participants into normal, borderline, and abnormal ranges.

In the present study, parents or legal guardians completed the proxy-report version of the SDQ on behalf of their children. The parent-proxy version was selected based on three primary methodological considerations: first, it is the standard and recommended version for the broader 4–17 age range under the established SDQ scoring protocols (17); second, since the sample included participants as young as 10 years of age, utilizing a parental report ensures reliable completion and minimizes potential floor or ceiling artifacts that can arise from self-report measures in early adolescence; and third, given that pediatric Long COVID is characterized by fluctuating symptomatology which may temporarily distort a child's self-assessment on any single day, parental observation offers a more stable and broader temporal perspective of the participant's emotional and behavioral functioning. However, the potential limitations of proxy-reports regarding subjective internalizing symptoms are acknowledged.

In addition, contextual functioning variables were collected through the online questionnaire as exploratory indicators of the condition's impact on daily life in developmentally relevant domains. These consisted of eight *ad hoc* items designed to evaluate: (1) school attendance since Long COVID onset (measured on a Likert-type scale: always, frequently, rarely, or not at all); (2) academic grade repetition due to illness-related absenteeism (yes/no); (3) loss of interest in previously enjoyed extracurricular or leisure activities (yes/no); (4) difficulties relating to peers (never, sometimes, frequently, or always); (5) sleep quality (good/very good, regular, or poor/very poor); (6) psychological changes since Long COVID onset (yes/no); (7) currently receiving psychological support (yes/no); and (8) self-perception (positive/very positive, neutral, or negative). While these items do not constitute a psychometrically validated scale, they represent directly observable and clinically relevant indicators developed based on commonly reported functional challenges in pediatric Long COVID.

Independent variables included demographic and clinical characteristics potentially associated with functional outcomes. These comprised age, sex, and area of residence (rural, semi-urban, or urban), as well as clinical variables such as number of SARS-CoV-2 infections, duration of persistent symptoms (in months), perceived symptom severity assessed using a numerical rating scale from 0 to 10 -where 0 indicated no perceived symptom burden and 10 indicated the maximum perceived severity-, and presence of pre-existing diagnoses. Information regarding type and frequency of treatments received (pharmacological and non-pharmacological) was also collected. In addition, perceived stress levels and recent emotional experiences were included as contextual variables. These items were developed *ad hoc* based on clinical relevance and commonly reported functional challenges in pediatric Long COVID. Sociodemographic characteristics were recorded to provide a comprehensive description of the sample.

### 2.4 Procedure

Data collection took place between March and July 2025 through an online questionnaire developed using Microsoft Forms® under the institutional license of Universidad Rey Juan Carlos. The survey included sociodemographic and clinical questions, contextual functioning items (school absences, grade repetition, withdrawal from extracurricular activities), and the standardized assessment instruments (WeeFIM and SDQ).

The survey link was distributed through collaborating patient associations and support networks to families meeting the inclusion criteria. Participation was voluntary and no financial incentives were provided. Electronic informed consent was obtained from parents or legal guardians prior to participation, and adolescents provided assent when appropriate. All data were anonymized using coded identifiers and handled in accordance with institutional ethical standards and applicable data protection regulations (Spanish Organic Law 3/2018).

### 2.5 Statistical analysis

Prior to inferential analysis, normality of distribution was assessed using the Shapiro–Wilk test, given the small sample size. Results indicated non-normal distribution for several variables, supporting the use of non-parametric tests. Descriptive statistics (means, standard deviations, frequencies, and percentages) were calculated for all variables.

To explore the relationship between variables, Spearman's rho was used to assess associations between continuous/ordinal variables and dichotomous variables coded as binary (0/1), given its robustness to non-normal distributions. Statistical significance was set at *p* < .05. All statistical analyses were conducted using IBM SPSS Statistics (version 29; IBM Corp., Armonk, NY, USA). Missing data were minimal across variables; cases with incomplete responses on key outcome measures were excluded from the corresponding analyses on a pairwise basis.

## 3 Results

### 3.1 Sociodemographic characteristics

The study included 27 participants with a mean age of 15.48 ± 2.31 years (range: 10–19). The sample was predominantly female (70.4%), with 25.9% male and 3.7% preferring not to specify. Most participants resided in semi-urban (48.1%) or urban (40.7%) environments. The mean age of parents or legal guardians was 48.22 ± 3.88 years (range: 41–57), with healthcare or social healthcare being the most common profession (33.3%). The majority of families had a medium socioeconomic level (88.9%) and did not receive financial aid (88.9%) (See Table 1).

**Table 1:** Sociodemographic characteristics of the sample (*n* = 27).

Descriptive statistics M ± SD (min–max)	Full sample (*n* = 27)
Age (years)	15.48 ± 2.31 (10–19)
Parent age (years)	48.22 ± 3.88 (41–57)
No. of infections	2.00 ± 0.96 (1–4)
Duration of persistent symptoms (months)	51.43 ± 11.08 (30–68)
Frequencies *n* (%)	Full sample (*n* = 27)
Sex	
Male	7 (25.9)
Female	19 (70.4)
Prefer not to say	1 (3.7)
Place of residence	
Rural	3 (11.1)
Semi-urban	13 (48.1)
Urban	11 (40.7)
Living arrangement	
Both parents	22 (81.5)
Mother only	5 (18.5)
Parent profession	
Healthcare or social healthcare	9 (33.3)
Non-healthcare higher education	7 (25.9)
Technical or mid-level professions	7 (25.9)
No formal studies	3 (11.1)
Socioeconomic status	
Low	1 (3.7)
Medium	24 (88.9)
High	2 (7.4)
Receives financial aid	
Yes	3 (11.1)
No	24 (88.9)
Prior diagnosis	
Yes	9 (33.3)
No	18 (66.7)

### 3.2 Analysis of persistent symptoms

**Figure 1:**
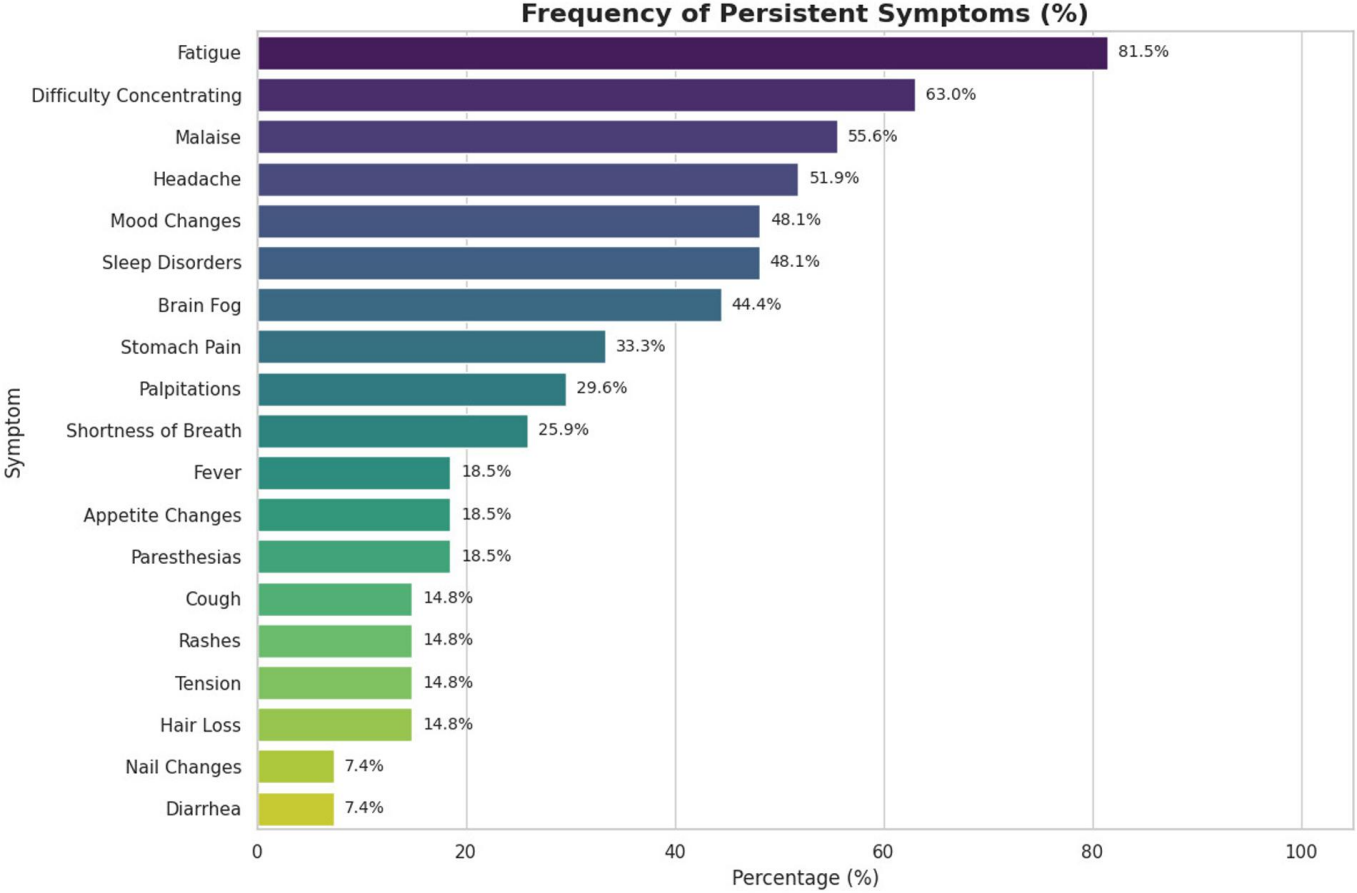
Frequency of persistent symptoms.

The analysis of persistent symptomatology within the study population indicates a high prevalence of various clinical manifestations, as depicted in Figure 1. The most frequent symptom reported was fatigue, affecting a substantial majority of participants (81.5%). This was followed by significant cognitive and neurological manifestations, including difficulty concentrating (63.0%), malaise (55.6%), and headache (51.9%), each present in more than half of the cohort.

Other symptoms with notable prevalence rates approaching 50% included mood changes (48.1%), sleep disorders (48.1%), and brain fog (defined as a subjective experience of cognitive sluggishness, mental fatigue, and difficulty concentrating) (44.4%).

Less frequent symptoms, yet still relevant within the clinical spectrum, included fever, appetite changes, and paresthesias, each occurring in 18.5% of cases. The least prevalent symptoms identified were cough, rashes, tension, and hair loss (all at 14.8%), while nail changes and diarrhea were reported by 7.4% of the sample. See Figure 1.

Seven out of the 19 symptoms assessed exceeded the 40% prevalence threshold.

### 3.3 Analysis contextual functioning variables

To complement the standardized assessments, contextual functioning data were collected through the online questionnaire across school, recreational, and social domains. Results are summarized in Table 2. Findings revealed substantial real-world impact across all domains. Only 18.5% of participants attended school regularly since the onset of Long COVID symptoms, and 11.1% had repeated an academic year due to illness-related absenteeism. The vast majority (85.2%) reported loss of interest in previously enjoyed activities. Psychological changes since onset were reported by 81.5% of families, yet 44.4% were not receiving any psychological support at the time of assessment.

**Table 2:** Contextual functioning variables: school, recreational, and social domains (*n* = 27).

Variable	*n* 27 (%)
School attendance since Long COVID onset	
Always	5 (18.5)
Frequently	13 (48.1)
Rarely	8 (29.6)
Not at all	1 (3.7)
Grade repetition due to absenteeism	
Yes	3 (11.1)
No	24 (88.9)
Loss of interest in previously enjoyed activities	
Yes	23 (85.2)
No	4 (14.8)
Difficulties relating to peers	
Never	11 (40.7)
Sometimes	11 (40.7)
Frequently	2 (7.4)
Always	3 (11.1)
Sleep quality	
Good/Very good	5 (18.5)
Regular	13 (48.1)
Poor/Very poor	9 (33.3)
Psychological changes since Long COVID onset	
Yes	22 (81.5)
No	5 (18.5)
Currently receiving psychological support	
Yes	15 (55.6)
No	12 (44.4)
Self-perception	
Positive/Very positive	7 (25.9)
Neutral	11 (40.7)
Negative	9 (33.3)

### 3.4 Analysis of functional independence and emotional and behavioral difficulties

The functional independence of the subjects was evaluated using the WeeFIM instrument across three primary domains: self-care, mobility, and cognition. The results indicate that the study population maintains a high level of functional independence, as the mean scores in all categories are situated close to the maximum possible values of the scale (Table 3). The high average scores (e.g., a total mean of 114.56 out of 126) suggest that participants generally perform daily activities with minimal to no assistance despite the presence of persistent symptoms.

**Table 3:** Descriptive analysis of functional independence measured with the WeeFIM and strengths and difficulties questionnaire (SDQ).

Standardized Assessment Scales M ± DS (min-max.)	
WeeFIM Total	114.56 ± 20.71 (18–126)
WeeFIM self-care	39.48 ± 7.36 (6–42)
WeeFIM mobility	33.26 ± 5.86 (5–35)
WeeFIM cognition	41.81 ± 8.77 (7–49)
SDQ Total	12.07 ± 5.04 (2–23)
SDQ emotional symptoms	5.59 ± 2.34 (1–10)
SDQ conduct problems	1.07 ± 1.35 (0–4)
SDQ hyperactivity	2.93 ± 2.18 (0–8)
SDQ peer relationship problems	2.48 ± 1.88 (0–6)
SDQ prosocial	8.96 ± 1.5 (5–10)

Regarding emotional and behavioral difficulties, the SDQ results indicated that participants had a total difficulties score of 12.07 ± 5.04, within the normal range. The prosocial behavior scale showed high scores 8.96 ± 1.5, reflecting strong social competencies. Regarding the subscales for emotional symptoms, conduct problems, hyperactivity, and peer relationship problems, the means were 5.59 ± 2.34, 1.07 ± 1.35, 2.93 ± 2.18, and 2.48 ± 1.88, respectively. These findings suggest that, overall, no significant behavioral problems were observed, although slightly elevated levels of emotional symptoms were present, while hyperactivity and peer relationship difficulties were moderate and did not indicate clinically significant concerns (see Table 3).

### 3.5 Correlation analysis of psychometric variables and somatic symptomatology

**Figure 2:**
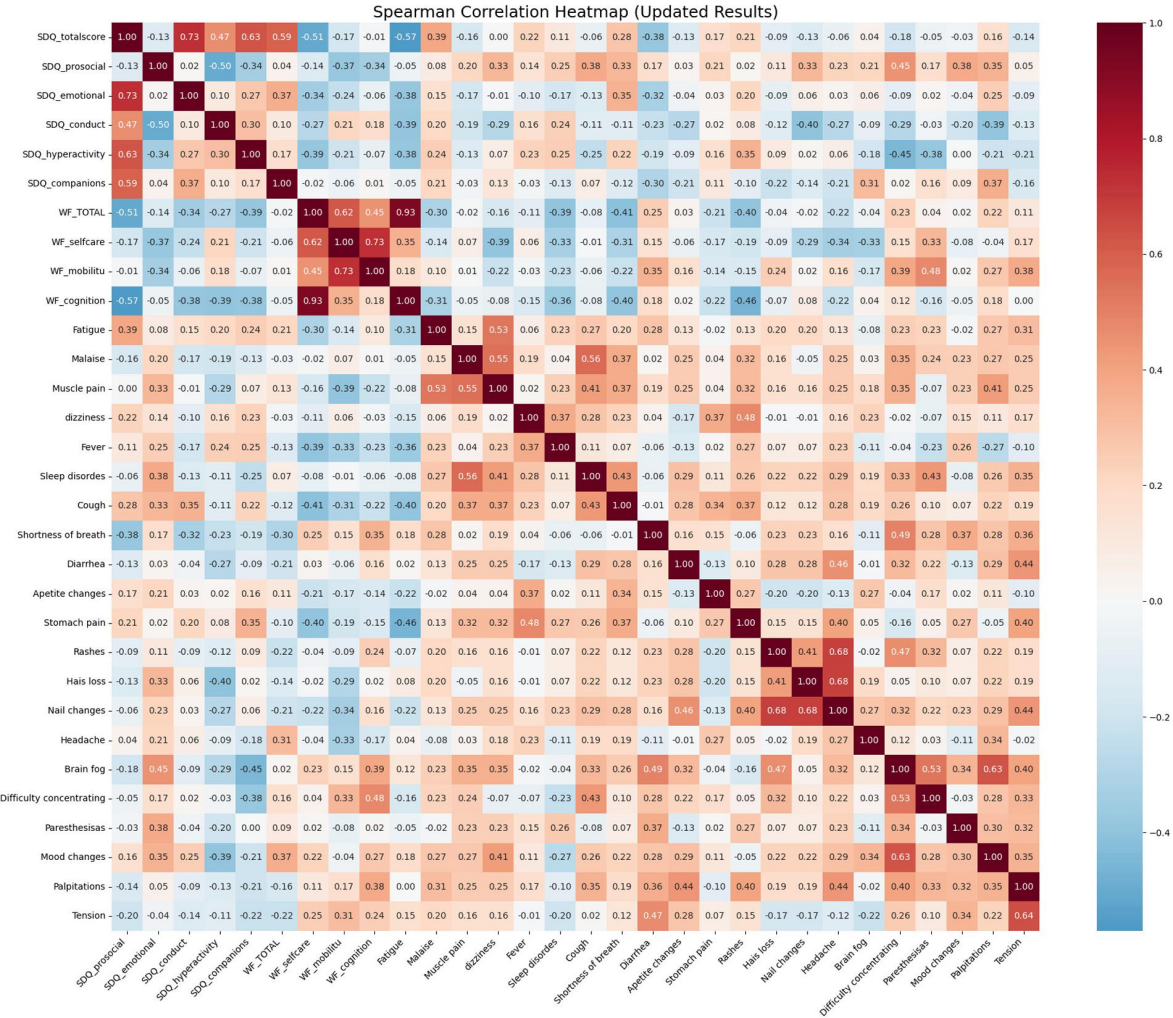
Heatmap spearman.

The Spearman correlation analysis revealed statistically significant associations between mental health dimensions, cognitive functioning, and reported physical symptomatology (Figure 2). A moderate-to-strong negative correlation was identified between the total score of the Strengths and Difficulties Questionnaire SDQ total score and cognitive functioning (WeeFim cognition score rho = −0.570, *p* = 0.0019), suggesting that an increase in psychopathological difficulties is associated with lower perceived performance in cognitive processes. Within the internal structure of the SDQ, the total score showed the highest covariance with the emotional symptoms dimension (rho = 0.730, *p* < 0.001) and hyperactivity (rho = 0.626, *p* < 0.001), establishing these as the primary determinants of the global difficulty construct in this sample.

Regarding persistent symptomatology, the phenomenon of “brain fog” demonstrated a robust positive association with mood disturbances (rho = 0.630, *p* < 0.001) and concentration deficits (rho = 0.532, *p* = 0.0043). In the somatic-vegetative domain, significant correlations were found between palpitations and subjective tension (rho = 0.643, *p* < 0.001), as well as between malaise and sleep disturbances (rho = 0.564, *p* = 0.0022), the latter also being linked to muscle pain (rho = 0.414, *p* = 0.0316). Finally, a pattern of dermatological convergence was observed, where nail changes exhibited strong correlations with hair loss and rash (rho = 0.678, *p* < 0.001), indicating a concurrent systemic manifestation of these alterations.

## 4 Discussion

### 4.1 Overview of findings

The present study examined functional independence, emotional functioning, and symptom burden in a sample of children and adolescents with Long COVID, integrating standardized assessment with contextual indicators of daily functioning. Results show a heterogeneous but consistently high symptom burden (particularly fatigue, cognitive difficulties, and mood-related symptoms) alongside largely preserved basic functional independence (WeeFIM).

Emotional functioning (SDQ) revealed a predominantly internalizing profile, with emotional symptoms contributing most to overall difficulties. Correlation analysis indicated a negative association between psychosocial difficulties and cognitive functioning, as well as a coherent cluster linking brain fog, mood, and concentration problems. The following subsections discuss these findings in the context of the existing literature and their implications for clinical practice and future research. This pattern reinforces the importance of examining potential discrepancies between basic functional independence and real-life functional impact in pediatric Long COVID.

### 4.2 High symptom burden with preserved functional independence: a pediatric resilience pattern?

The symptom profile observed in this sample aligns with patterns reported in prior pediatric Long COVID studies. Heidar Alizadeh et al. (19), in a comprehensive umbrella review, identified fatigue and cognitive symptoms as the most frequently reported manifestations across pediatric cohorts worldwide, with female sex and older age as recurring associated factors—similar to the predominantly female and adolescent composition of the present sample (19). Similarly, the CLoCk cohort study in England, which followed over 3,000 adolescents, documented persistent fatigue, cognitive difficulties, and emotional symptoms in a significant proportion of SARS-CoV-2-positive young people three months post-infection (11). Notably, 88.9% of participants in the present sample had experienced persistent symptoms for more than 24 months (mean 51.43 months), placing this cohort at the more chronic end of the spectrum compared to most published studies. This chronicity is consistent with prospective evidence documenting sustained symptom burden over at least twelve months in pediatric Long COVID cohorts (3). Consistent symptom profiles (including fatigue, cognitive difficulties, and mood disturbances) have also been documented in large European population-based cohorts, with prevalence estimates varying widely depending on methodology and follow-up duration (20).

An important interpretive caveat concerns the chronic and severe nature of the present sample. The mean symptom duration of 51.43 months (range: 30–68 months) places this cohort at the more persistent end of the pediatric Long COVID spectrum, considerably exceeding the follow-up periods of most published studies and differing from cohorts that captured earlier or less chronic disease presentations. This level of chronicity may reflect, at least in part, a selection effect attributable to the recruitment strategy: families engaged with patient associations and support networks are likely to represent those with greater symptom burden and longer illness trajectories (21). Accordingly, estimates of symptom prevalence, functional impact, and emotional burden derived from this sample should not be generalized to pediatric Long COVID populations at earlier stages of the condition or to those identified through population-based or clinical surveillance approaches. The findings are best understood as characterizing a subgroup with particularly persistent and severe presentations and should be interpreted accordingly.

What stands out in the present findings is the contrast between the high symptom load and the significant functional autonomy in daily activities. Near-maximal WeeFIM scores for self-care, mobility, and cognition suggest that this population maintains substantial independence in routine tasks. This may reflect a key difference between pediatric and adult Long COVID: children and adolescents, supported by family and developmental resilience, can sustain functional routines despite significant symptoms. This aligns with neuropsychological frameworks emphasizing the plasticity and compensatory capacity of the developing nervous system in chronic conditions (4).

However, caution is warranted in reading preserved WeeFIM scores as evidence of intact everyday functioning. The contextual functioning data collected in this study stand in marked contrast to these near-ceiling scores: only 18.5% of participants attended school regularly since the onset of Long COVID, 11.1% had repeated an academic year due to illness-related absenteeism, and 85.2% had withdrawn from recreational activities they previously enjoyed. These figures illustrate a dissociation between independence in basic structured activities and real-world functioning in higher-demand, meaningful contexts (6, 7). Framed directly within the International Classification of Functioning, Disability and Health (ICF), this finding represents a stark divergence between a child's *capacity*—what they can execute in a standardized, controlled environment (captured by the WeeFIM)—and their actual *performance* in real-life contexts (captured by the contextual variables). The WeeFIM, designed to detect assistance needs across a broad severity spectrum, operates primarily as a measure of basic activity capacity; it lacks the sensitivity and the specific performance qualifiers required to capture the fluctuating, post-exertional, and higher-order participation restrictions characteristic of pediatric Long COVID (8, 10).

### 4.3 The challenge of assessing everyday functioning in pediatric long COVID: a methodological gap in the field

A key finding of this study is the methodological challenge in evaluating the impact of Long COVID on everyday functioning in children and adolescents. Existing functional assessment tools in pediatric rehabilitation were developed primarily for populations with stable disabilities and have not been validated for post-viral conditions like Long COVID, which present with fluctuating symptoms. While tools like PEM-CY and CAPE/PAC provide useful frameworks for assessing restriction in school, recreational, and community activities, their applicability and sensitivity to this population are not yet established (8). The symptom profile of pediatric Long COVID (including post-exertional malaise, fluctuating fatigue, and neurocognitive dysfunction) closely overlaps with that of myalgic encephalomyelitis/chronic fatigue syndrome (ME/CFS), which has been documented in children as young as 11 years following SARS-CoV-2 infection and which is also associated with substantial reductions in educational and daily functioning not adequately captured by standard independence measures (22).

Faced with this gap, the present study used a structured questionnaire to collect contextual functioning variables, exploring the real-world impact of Long COVID. These included school absences, academic grade repetition, and withdrawal from extracurricular activities. By examining these items collectively, a clear pattern of multidimensional restriction emerges. The high rates of school absenteeism and severe withdrawal from leisure activities do not exist in isolation; rather, they co-occur with substantial secondary challenges, such as poor sleep quality and self-reported difficulties relating to peers. Discussing these variables together reinforces the theoretical argument that pediatric Long COVID dynamically compromises the child's entire ecosystem. Preserved basic physical autonomy (WeeFIM) creates a false impression of recovery, whereas the contextual data explicitly expose how persistent somatic symptoms disrupt fundamental developmental pillars—education, socialization, and rest—thereby demonstrating a profound dissociation between basic capacity and actual real-life performance.

From the perspective of Occupational Science and the Person-Environment-Occupation (PEO) model, this multidimensional restriction reflects a critical disruption in the dynamic transaction between the adolescent (the person experiencing fluctuating symptoms), their everyday contexts (the school and social environment), and their meaningful activities (the occupations) (23). When severe fatigue and cognitive symptoms collide with the rigid, high-demand expectations of academic and social environments, the fit between person, environment, and occupation collapses. Consequently, the drastic withdrawal from school and leisure identified in our sample represents a state of occupational deprivation rather than a mere accumulation of symptom burden, as these children are systematically barred from engaging in the very activities that drive healthy neurodevelopment and identity construction.

This finding highlights the need for the development and validation of functional assessment tools tailored to pediatric Long COVID, addressing symptom fluctuation, post-exertional malaise, and varying impacts based on context and activity demand. Until such tools are available, combining standardized measures with systematically collected contextual functioning variables provides a more comprehensive approach than relying on conventional functional assessments alone.

### 4.4 Emotional functioning: internalizing profile and clinical significance

Although the group-level SDQ total score was within the normal range, the slightly elevated emotional symptoms subscale and internalizing difficulties warrant clinical attention. The predominance of emotional over conduct or hyperactivity issues aligns with the internalizing profile seen in pediatric Long COVID. Noij et al. (12), in a multicohort study in the Netherlands, found that children with Long COVID had significantly worse health-related quality of life and higher levels of anxiety, depression, and sleep disturbance compared to those with other chronic conditions and general pediatric population (12). This underscores the central role of emotional symptoms in pediatric Long COVID.

The SDQ analysis in this sample revealed that emotional symptoms most strongly correlated with the total score, followed by hyperactivity/inattention, suggesting that internalizing and attentional difficulties drive overall psychosocial challenges. Notably, prosocial behavior remained intact, which may indicate that relational motivation persists even amidst emotional distress, potentially offering a therapeutic resource.

An important interpretive consideration is the overlap between Long COVID symptoms (e.g., fatigue, malaise, sleep disturbance) and emotional well-being measures. The WHO pediatric case definition highlights the challenge of distinguishing between somatic, cognitive, and psychological manifestations in this population (1). The CLoCk study further noted that some mood and somatic symptoms are also present in SARS-CoV-2-negative adolescents, emphasizing the need to view emotional findings within the broader context of pandemic-related disruptions, such as school closures, social isolation, and family stress, rather than attributing all psychosocial difficulties solely to the infection (11, 24). Critically, the present study lacks a comparison group of SARS-CoV-2-negative or test-negative children, which means it is not possible to determine whether the observed levels of emotional distress, school absenteeism, and reduced participation are specifically attributable to Long COVID or whether they partly reflect broader sequelae of the pandemic period experienced by pediatric populations more generally. Pandemic-related disruptions—including prolonged school closures, social isolation, and family-level stress—are plausible confounders that cannot be ruled out in the absence of a control group. Consequently, the emotional and participation findings reported here should not be interpreted as Long COVID-specific effects; they describe characteristics of this sample but cannot establish causal or differential attribution. Recent large-cohort evidence further supports this interpretive caution, demonstrating that difficulties in children and adolescents with Long COVID must be evaluated against population-level pandemic baseline data to isolate condition-specific effects (25).

### 4.5 The symptom cognition emotion triad: interconnected vulnerability domains

The correlational analysis revealed a significant negative association between overall psychosocial difficulty (SDQ total score) and WeeFIM cognition scores (rho = −0.570, *p* = 0.0019), suggesting that greater emotional and behavioral challenges are linked to poorer perceived cognitive performance in daily life. This finding aligns with the understanding that emotional dysregulation and psychological burden can impair cognitive functions such as attention, working memory, and executive function (26, 27), and is consistent with recent evidence in pediatric Long COVID specifically showing that anxiety and depression are significant predictors of everyday functional limitations, even when controlling for other symptoms (28).

Indeed, the brain fog–mood–concentration triad identified in this sample illustrates this interconnectedness precisely. Brain fog correlated strongly with mood disturbances (rho = 0.630, *p* < 0.001) and concentration issues (rho = 0.532, *p* = 0.0043), forming a cluster of symptoms where each component amplifies the others. This triad can be robustly understood through a comprehensive biopsychosocial framework, which conceptualizes post-viral conditions not merely as isolated physical or psychological sequelae, but as a dynamic, bidirectional feedback loop. Mechanistically, this aligns with established neuro-immune and psychophysiological models of myalgic encephalomyelitis/chronic fatigue syndrome (ME/CFS), where persistent low-grade neuroinflammation or dysautonomia (biological factors) directly disrupts executive function and concentration (cognitive factors), subsequently generating severe emotional distress and anxiety regarding academic decline (psychosocial factors). In the somatic domain, the link between malaise and sleep disturbance (rho = 0.564, *p* = 0.0022), as well as the connection between sleep disturbance and muscle pain (rho = 0.414, *p* = 0.0316), further reflects this biopsychosocial cycle. Disrupted sleep impairs central pain processing and immune regulation, which in turn exacerbates physical malaise, creating a self-perpetuating loop of functional limitation where biological dysfunction and psychological strain are mutually reinforcing (16, 29).

These symptom clusters have clear implications for treatment. Focusing on one component in isolation—for example, targeting fatigue without addressing sleep quality or emotional distress is unlikely to be effective. An integrated, multicomponent approach that simultaneously addresses physical symptom management, cognitive support, and emotional regulation is more likely to lead to meaningful functional improvements (5, 30). This approach aligns with the biopsychosocial framework increasingly recommended in pediatric Long COVID clinical guidelines (1, 29).

### 4.6 Clinical, rehabilitation, and educational implications

The findings of this study carry practical implications across three interconnected levels: clinical assessment, rehabilitation, and school reintegration.

In terms of clinical assessment, the study highlights that traditional functional independence measures like WeeFIM, though useful, are insufficient to fully capture the impact of Long COVID in pediatric populations. The presence of high symptom burden, particularly fatigue, cognitive difficulties, and mood disturbances, alongside largely preserved functional independence, suggests that these tools fail to capture the real-world consequences of Long COVID. Contextual factors such as school absences, academic struggles, and withdrawal from extracurricular activities further underscore the need for more comprehensive assessment protocols. These should incorporate both standardized measures and systematically collected indicators of daily functioning, emotional well-being, and contextual challenges. This calls for the development of assessment tools specifically validated for pediatric Long COVID, sensitive to the fluctuation of symptoms and varying impacts across different contexts and activity demands.

At the rehabilitation level, the findings support a multicomponent, function-centered approach. Addressing not only physical symptoms but also cognitive difficulties and emotional regulation is crucial for improving overall function. Given the symptom profile, energy management strategies such as pacing and post-exertional malaise prevention are particularly important (29). By anchoring these interventions within occupation-based frameworks, occupational therapy is uniquely positioned to address the occupational imbalances identified in this population. Rather than focusing strictly on impairment reduction, occupational therapy utilizes its core theoretical focus on the transaction between person, environment, and occupation to design comprehensive adaptations. This includes grading activity demands to match the child's fluctuating energy envelope, modifying physical and cognitive environments, and facilitating structured, low-demand avenues for social re-engagement. This comprehensive approach ensures that rehabilitation targets not just physical independent capacity, but the active restoration of real-world participation and quality of life (7).

In terms of school reintegration, the study underscores the importance of individualized academic accommodations. MacLean et al. (31) documented, through qualitative analysis, that children and young people with Long COVID and their families consistently emphasize the critical importance of being believed, understood, and supported through flexible, coordinated school adjustments (31). Provisions such as phased return, reduced timetables, rest periods, and cognitive load adjustments (developed in partnership between healthcare providers, families, and educational institutions) may be decisive in preventing academic disengagement, social isolation, and the consequent amplification of emotional burden.

### 4.7 Limitations and future directions

Several limitations must be considered in interpreting these findings. First, the sample size (*n* = 27), while acceptable for an exploratory cross-sectional study, limits statistical power and generalizability. In addition, the recruitment strategy relies on convenience sampling via anonymous online distribution through patient associations, which did not permit tracking of the number of families who received the survey invitation or computing a formal response rate. As a result, it is not possible to report the ratio of families approached to those enrolled, nor to assess the representativeness of respondents relative to those who did not participate. Moreover, recruitment through specialized patient associations may have introduced selection bias, potentially inflating the prevalence of certain clinical manifestations due to higher symptom severity or increased motivation to participate. This likely favored the inclusion of more symptomatic and highly engaged families, which may overestimate symptom burden and functional impact relative to unselected pediatric Long COVID populations. Furthermore, the mean symptom duration of 51.43 months (range: 30–68 months) indicates that this cohort represents the more severe and persistent end of the pediatric Long COVID spectrum. Findings should therefore not be generalized to less chronic presentations or to cohorts identified through population-based or clinical surveillance methods, and should be understood as characterizing a particularly affected subgroup rather than the broader pediatric Long COVID population.

Second, the cross-sectional design prevents conclusions about causality or temporal directionality. The correlations observed between emotional symptoms and cognitive functioning align with bidirectional models, but longitudinal studies are necessary to determine whether emotional difficulties precede, result from, or co-evolve with functional limitations. The broader project, including a six-month follow-up, will provide further insight into these outcomes over time.

Third, the field lacks validated instruments to assess real-life functioning in pediatric Long COVID. Existing participation measures in pediatric rehabilitation are not adapted for post-viral conditions with fluctuating symptoms. In this study, contextual functioning variables (e.g., school absences, grade repetition) were explored, but these are not psychometrically validated. Developing instruments sensitive to symptom fluctuation and context-specific impacts is a key priority for future research.

Fourth, the WeeFIM may show ceiling effects in this population due to its limited sensitivity to the fluctuating, effort-dependent functional limitations of Long COVID. As noted in the Variables and Instruments section, near-ceiling scores were anticipated *a priori* based on prior literature and constitute a methodological rationale for the study's contextual approach rather than an unexpected finding. Additionally, reliance on self-report and proxy-report measures introduces the potential for memory bias and inconsistencies in symptom perception between parents and children. Specifically, while the use of the parent-proxy version of the SDQ was methodologically justified to optimize reliability and maintain a stable temporal overview, it represents a notable limitation as parent ratings may not fully align with or completely capture the child's own subjective, internal emotional experience. This potential discordance between parental observation and adolescent self-perception must be taken into account when interpreting the internalizing profile identified in this cohort.

Lastly, the absence of a control group without Long COVID limits the ability to isolate Long COVID-specific effects from broader developmental, psychosocial, or pandemic-related factors. This is a critical limitation: it is not possible to establish that the observed emotional difficulties, school absenteeism, or reduced participation in recreational activities are specifically caused by or attributable to Long COVID, as opposed to reflecting broader pandemic-era sequelae affecting pediatric populations more generally. Without a test-negative or population-based comparison group, causal inference regarding Long COVID-specific effects on emotional functioning and daily participation cannot be drawn. Future studies should include test-negative or population-based comparison groups, following the methodology of the CLoCk study (Stephenson et al., 2022), to strengthen causal inference and estimate the attributable burden of Long COVID. Large-cohort studies such as that by underscore the importance of comparison groups to isolate Long COVID-specific effects from broader pandemic sequelae (25).

Future research should focus on longitudinal cohorts with adequate follow-up, the development of validated functional assessment tools specific to pediatric Long COVID, multimethod assessments, and the exploration of sex- and age-related moderators. Additionally, the design of standardized rehabilitation outcome measures and international collaboration, such as the RECOVER pediatric initiative (19), will be crucial for building a robust evidence base to guide clinical practice and health policy.

## 5 Conclusion

This study provides preliminary but clinically meaningful evidence on the multidimensional impact of Long COVID in children and adolescents. Despite a high and heterogeneous symptom burden—including fatigue, cognitive difficulties, and mood disturbances—participants largely maintained functional independence in basic daily activities, challenging the assumption that standard measures like the WeeFIM adequately capture the real-life impact of the condition. Contextual variables including school absenteeism, grade repetition, and withdrawal from recreational activities reveal a broader functional impact that conventional tools fail to detect.

Cognitive functioning emerged as a particularly vulnerable domain, evidenced by the negative association between psychosocial difficulty and cognitive performance and the coherent brain fog–mood–concentration cluster. Emotional and cognitive difficulties are central axes of the functional impact of pediatric Long COVID, with direct implications for daily life, academic performance, and social engagement. At the same time, the relative preservation of functional independence and prosocial behavior suggests a degree of developmental resilience that may serve as a therapeutic resource in clinical practice. It should be noted, however, that the absence of a control group precludes causal attribution of these findings specifically to Long COVID, as pandemic-related factors may contribute to the observed patterns.

These findings underscore the need for assessment and intervention approaches that extend beyond basic functional independence to encompass real-life functioning, emotional well-being, and cognitive performance. Coordinated, multidisciplinary care and the development of validated Long COVID-specific assessment tools remain key priorities for advancing both clinical practice and the field.

## Data Availability

The datasets presented in this article are not readily available because they contain sensitive information from pediatric participants and are protected under applicable data protection and confidentiality regulations. Anonymized data may be made available by the corresponding author upon reasonable request and subject to ethical approval. Requests to access the datasets should be directed to sleon@unizar.es.
